# Genetic Loci Governing Androgenic Capacity in Perennial Ryegrass (*Lolium perenne* L.)

**DOI:** 10.1534/g3.117.300550

**Published:** 2018-04-06

**Authors:** Rachel F. Begheyn, Steven A. Yates, Timothy Sykes, Bruno Studer

**Affiliations:** Molecular Plant Breeding, Institute of Agricultural Sciences, ETH Zurich, 8092 Zurich, Switzerland

**Keywords:** Anther culture (AC), Doubled haploid (DH), Perennial ryegrass (*Lolium perenne* L.), Genome-wide association study (GWAS), Microspore embryogenesis (ME), Multiparental populations

## Abstract

Immature pollen can be induced to switch developmental pathways from gametogenesis to embryogenesis and subsequently regenerate into homozygous, diploid plants. Such androgenic production of doubled haploids is particularly useful for species where inbreeding is hampered by effective self-incompatibility systems. Therefore, increasing the generally low androgenic capacity of perennial ryegrass (*Lolium perenne* L.) germplasm would enable the efficient production of homozygous plant material, so that a more effective exploitation of heterosis through hybrid breeding schemes can be realized. Here, we present the results of a genome-wide association study in a heterozygous, multiparental population of perennial ryegrass (n = 391) segregating for androgenic capacity. Genotyping-by-sequencing was used to interrogate gene- dense genomic regions and revealed over 1,100 polymorphic sites. Between one and 10 quantitative trait loci (QTL) were identified for anther response, embryo and total plant production, green and albino plant production and regeneration. Most traits were under polygenic control, although a major QTL on linkage group 5 was associated with green plant regeneration. Distinct genetic factors seem to affect green and albino plant recovery. Two intriguing candidate genes, encoding chromatin binding domains of the developmental phase transition regulator, Polycomb Repressive Complex 2, were identified. Our results shed the first light on the molecular mechanisms behind perennial ryegrass microspore embryogenesis and enable marker-assisted introgression of androgenic capacity into recalcitrant germplasm of this forage crop of global significance.

In contrast to animals, plant cellular differentiation (cell fate) is both flexible and reversible (Walbot and Evans 2003). In immature male gametophytic cells, a totipotent state can be induced through the application of a stress treatment. Subsequent de-differentiation of such cells into the embryogenic pathway may then be stimulated via their cultivation under suitable *in vitro* conditions. This process, known as microspore embryogenesis (ME) or androgenesis, ultimately results in the recovery of haploid or, via spontaneous or induced chromosome doubling, diploid completely homozygous individuals (Seguí-Simarro and Nuez 2008). Segregating populations of male gametophytes can thus be transformed into doubled haploids (DHs) in a single generation. These are of great value to fundamental research as well as plant breeding (Forster *et al.* 2007). The practical utility of androgenesis, however, ultimately depends on the efficient production of large numbers of microspore-derived embryos capable of regeneration into green, fertile plants.

The optimum stress and *in vitro* culture conditions for successful androgenesis are highly species and genotype-dependent (Seguí-Simarro 2010; Dwivedi *et al.* 2015). Through decades of empirical research, highly effective isolated microspore culture (IMC) protocols have been developed for barley (*Hordeum vulgare* L.), rapeseed (*Brassica napus* L.) and tobacco (*Nicotiana* spp.). Unfortunately, many economically (Solanaceae, fruit trees) and academically (*Arabidopsis*) important species remain recalcitrant (Seguí-Simarro 2015). In monocots, and grasses in particular, high rates of albinism further limit androgenic efficiency (Kumari *et al.* 2009). Apart from efforts aimed at establishing which external factors are critical for efficient androgenesis, attempts to uncover the genetic factors controlling ME and plant regeneration have been made.

In many cereal crops, linkage mapping studies have identified chromosomal regions associated with traits related to androgenesis. Quantitative trait loci (QTL) related to embryo production, for example, have been reported in wheat (*Triticum aestivum* L.) (Agache *et al.* 1989), barley (Manninen 2000) and triticale (× *Triticosecale* Wittm.) (González *et al.* 2005; Krzewska *et al.* 2012). The combined effect of two QTL on barley chromosomes 5H and 6H explained 51% of variation in green plant recovery (Chen *et al.* 2007), although only one QTL on chromosome 3H was implicated in a different study (Muñoz-Amatriaín *et al.* 2008). Two regions on wheat chromosomes 1B and 7B explained 53% of the observed variation in albinism (Nielsen *et al.* 2015), QTL for which have also been reported in barley and triticale (Bregitzer and Campbell 2001; Krzewska *et al.* 2015). However, due to a lack of protocol uniformity, the diversity of material under study and the high variability inherent to tissue culture, consensus among these types of investigations is low (Bolibok and Rakoczy-Trojanowska 2006; Seldimirova and Kruglova 2015). In addition, genes underlying any of the reported QTL have not been identified.

Nevertheless, a number of candidate genes have been associated with high levels of ME and plant regeneration by means of gene expression experiments (reviewed in Hand *et al.* 2016). For example, expression of somatic embryogenesis receptor kinase (SERK) gene *SERK1*, and in some cases *SERK2*, was correlated with embryo production and plant regeneration in species such as *Arabidopsis*, rapeseed, maize (*Zea mays* L.) and wheat (Hu *et al.* 2005; Singla *et al.* 2008; Podio *et al.* 2014; Ahmadi *et al.* 2016; Seifert *et al.* 2016). Overexpression of the *APETALA 2* (*AP2*) transcription factor *BABYBOOM* (*BBM*), *WUSCHEL* (*WUS*) and *AGAMOUS*-like (*AGL*) genes, led to the production of ectopic somatic embryos in *Arabidopsis*, rapeseed and a number of monocot species and improved *in vitro* regeneration frequencies (Boutilier 2002; Muñoz-Amatriaín *et al.* 2009b; Lowe *et al.* 2016). Other examples of genes that may be associated with ME are the arabinogalactan-related *EARLY CULTURE ABUNDANT 1* (*ECA1*) (Vrinten *et al.* 1999), Polycomb Group (PcG) proteins including *FERTILIZATION INDEPENDENT ENDOSPERM* (*FIE*) (Hand *et al.* 2016), BURP-domain proteins like *BnBNM2* (Boutilier 2002; Tsuwamoto *et al.* 2007; Joosen *et al.* 2007; Malik *et al.* 2007) and the *LEAFY COTYLEDON* (*LEC*) family of transcription factors (Gruszczyńska and Rakoczy-Trojanowska 2011; Soriano *et al.* 2013; Elahi *et al.* 2016). Similar to linkage mapping studies, the use of different species, treatments and gene expression platforms as well as the complexity of the system under study, prohibit conclusive identification of the genes of greatest importance to successful androgenesis (Soriano *et al.* 2013).

Chromosomal regions or genes associated with androgenic capacity in the most widely grown forage species in temperate agriculture, perennial ryegrass (*Lolium perenne* L.), have not yet been identified. Previous studies concluded that perennial ryegrass’ androgenic capacity is under polygenic control, with distinct genetic factors influencing embryo production, plant regeneration and green or albino plant production (Olesen *et al.* 1988; Boppenmeier *et al.* 1989; Opsahl-Ferstad *et al.* 1994; Madsen *et al.* 1995; Begheyn *et al.* 2017). Additive and dominance effects may play a role in embryo and plant production, while green plant production involved dominance effects or the complementation of recessive beneficial alleles. Environmental rather than genetic factors may be the main cause of the high incidence of albinism exhibited by many genotypes (Begheyn *et al.* 2017).

In concert with recent efforts to move toward hybrid perennial ryegrass breeding, the potential of *in vitro* androgenesis for the efficient production of homozygous lines has been recognized (Arias Aguirre *et al.* 2011; Begheyn *et al.* 2016; Manzanares *et al.* 2016; Sykes *et al.* 2016). To overcome the problematic recalcitrance of most breeding germplasm, molecular marker-based introgression of beneficial alleles has been proposed (Halberg *et al.* 1990; Andersen *et al.* 1997). Therefore, the main objective of our study was to identify genetic loci associated with androgenic capacity in a multiparental perennial ryegrass population via a genome-wide association study (GWAS). In addition, we aimed at identifying potential causal genes that may provide clues to the molecular mechanisms behind ME and plant regeneration in this important member of the grass family.

## Materials and methods

### Plant material and anther culture procedure

A detailed description of most of the plant material and the *in vitro* anther culture (AC) procedure used here can be found in Begheyn *et al.* (2017). Briefly, nine perennial ryegrass genotypes with distinct androgenic capacities were pair-crossed as part of a DH induction program at the DLF A/S research station in Store Heddinge, Denmark (Table S1). Eleven populations of pair-cross offspring were grown in 1 L soil filled pots in an unheated greenhouse in Lindau, Switzerland, vernalized and used as anther donors in 2015 and 2016. Spikes containing microspores in the late-uninucleate stage were harvested and subjected to a 4° cold stress treatment of 24-72 h in the dark. After surface sterilization, anthers were aseptically excised and cultured on an adapted 190-2 induction medium (Wang and Hu 1984) in a 90 mm Petri dish, incubated at 26° with a 16 h photoperiod. After six to eight weeks, macroscopic embryo-like structures (ELS) were transferred to the regeneration medium for shoot and root induction.

### Phenotypic data collection

To quantify androgenic responses of the anther donor genotypes to *in vitro* AC, eight phenotypic traits were recorded: (1) anther response as a percentage of anthers producing macroscopic ELS (hereafter ‘responding anthers’ or RA); (2) embryo production as the number of ELS per 100 anthers cultured (AC); (3) plant, (4) green plant and (5) albino plant production, recorded per 100 AC; and (6) plant, (7) green plant and (8) albino plant regeneration, recorded per 100 ELS cultured. In 2015, a total of 313 genotypes were investigated, while incomplete vernalization prior to 2016 resulted in 116 studied genotypes. A total of 78 genotypes were phenotyped in both years (Table S1; Begheyn *et al.* 2017).

### DNA extraction

Fresh leaf tissue of the anther donor plants was harvested for DNA extraction on a 96-well plate KingFisher Flex Purification System with KingFisher Pure DNA Plant Kits (Thermo Fisher Scientific, Waltham, MA, USA). Genomic DNA was visualized on a 1% agarose gel and quantified with a NanoDrop 8000 spectrophotometer (Thermo Fisher Scientific, Waltham, MA, USA).

### Genotyping-by-sequencing library preparation

Genotyping-by-sequencing (GBS) libraries were prepared by multiplexing single restriction enzyme digested genomic DNA using 192 unique 5-10 bp barcodes (Table S2), designed with the Deena Bioinformatics online GBS Barcode Generator (http://www.deenabio.com/nl/services/gbs-adapters) and synthesized by Microsynth (Balgach, Switzerland).

Per sample, a 20 µL *Pst*I digestion mixture was prepared, containing 10 µL DNA sample (10 ng µL^-1^), 1 µL *Pst*I (3.5 U µL^-1^), 2.5 µL barcoded adaptors (0.1 ng µL^-1^), 2.5 µL common adaptors (0.1 ng µL^-1^), 2 µL O buffer and 2 µL H_2_O. Samples were digested for 2 h at 37°. Ligation with T4 ligase, pooling of 96 samples and purification (Qiagen MinElute PCR Purification Kit; Qiagen, Hilden, Germany) were performed according to Elshire *et al.* (2011). Fragments were amplified in volumes of 50 µL, containing 5 µL DNA library, 0.25 µL DreamTaq DNA Polymerase (5 U µL^-1^), 5 µL 10× DreamTaq Buffer, 5 µL dNTPS (2 mM), 1 µL primers (10 µM; Table S2) and 33.75 µL H_2_O. Thermocycler steps were as follows: 72° for 5 min, 95° for 30 s, 21 cycles of 95° for 10 s, 65° for 30 s and 72° for 30 s, with a 5 min final extension at 72° (GeneAMP PCR System 9700; Thermo Fisher Scientific, Waltham, MA, USA). All enzymes and their associated buffers were purchased from Thermo Fisher Scientific. Purified (as above) fragments were visualized on a 2200 TapeStation (Agilent Technologies, Santa Clara, CA, USA) to check for presence of adapter dimers and confirm a majority fragment length of 200-400 bp. If adapter dimers were present, an Agencourt AMPure XP bead purification (Beckman Coulter Inc., Brea, CA, USA) was performed.

### GBS library sequencing

Two 192-plex and one 39-plex anther donor GBS libraries (423 genotypes in total) were sequenced using 126 bp single-end reads on three lanes of an Illumina HiSequation 2500 platform at the Functional Genomics Center Zurich, Switzerland.

### GBS data processing, read mapping and variant calling

Reads were de-multiplexed using sabre (https://github.com/najoshi/sabre) allowing one mismatch. Using Bash commands and custom Perl scripts, reads were trimmed to 100 bp and the frequency (counts) of unique sequences (tags) was summarized per pair-cross population. Unique tags were back-transformed to FASTQ format. Bowtie v0.12.7 (Langmead *et al.* 2009) with “–best–strata” and a maximum of two alignments “-m 2” was used to map the FASTQ files to the perennial ryegrass genome v1.0 (Byrne *et al.* 2015). Unmapped tags were filtered out using a custom Perl script, resulting in 141,775,689 (20.2% of de-multiplexed) mapped tags. The SAM files as well as the count files were further processed in R v3.3.3 (R Core Team 2017).

Numerical factors were set to constrain genotyping to reflect the ploidy level of the genotypes (2*n*) and the maximum allele number (four) for pair-cross populations. Cut off values of 100 for the minor allele frequency (MAF) and eight for the minimum allele count (minAC) were used. Unique position identifiers (Upos) were extracted from the SAM files by concatenating the direction (Flag), location (Ref) and position (Pos) data. Low coverage sites were eliminated by retaining only Upos with at least one tag greater than the MAF. From the resulting tags, only those occurring at a frequency greater than 5% were retained.

For genotype calling, all informative, polymorphic nucleotide sites (Isites) across the tags were identified and only informative tags (Itags) with Isites were retained. Two unique alleles at one Isite position were called as heterozygous, while the occurrence of a single allele at one Isite was called as homozygous if its count was greater than the minAC. Informative tags were excluded if the number of unique Isites was greater than the ploidy level, or if the allele number within an Isites was greater than the maximum allele number. Haplotypes were obtained by concatenating alleles at the Isites within each tag, if applicable.

### Genome-wide association mapping (GWAS)

Population structure was investigated using STRUCTURE v2.3.4 (Hubisz *et al.* 2009), GAPIT v2 (Lipka *et al.* 2012) as well as the hierarchical clustering hclust() (method = “ward.D”) and principal component analysis (PCA) prcomp() functions in R.

Itags were filtered using a MAF threshold of 10% and a minimum of 100 and 50 genotypes in 2015 and 2016, respectively (Figure S1). Since the phenotypic data did not, and could not be made to, fit the criteria for parametric testing (Begheyn *et al.* 2017), the non-parametric, rank-based Kruskal-Wallis (K-W) test was used to detect associations between each segregating haplotype (Itag) and the phenotypic traits (Kiviharju *et al.* 2004; Krzewska *et al.* 2012). For each of these K-W tests, 10,000 random permutations of the phenotypes were run. Associations were considered significant at a K-W LOD of 3.0 or higher and a permutation test threshold of 1%. Bonferroni corrected Dunn’s tests (*P* ≤ 0.05) were carried out *post hoc* to compare haplotypes’ trait values. All statistical analyses were performed using custom scripts in Rstudio v1.0.143 (RStudio Team 2015), running R v3.3.3 (R Core Team 2017). The R packages ggplot2 (Wickham 2009) and UpSetR (Lex *et al.* 2014) were used to generate the figures.

Scaffolds of the perennial ryegrass genome v1.0 (Byrne *et al.* 2015) containing significant Itags will hereafter be referred to as “significant scaffolds”.

### Positioning the significant scaffolds on the GenomeZipper

Significant scaffolds were compared against the genome sequences of *Brachypodium distachyon*, rice (*Oryza sativa* Japonica Group) and sorghum (*Sorghum bicolor* L.) using a BLASTN search (E ≤ 1e^-5^, sequence identity ≥ 85%, match length of ≥ 150 bp). Matches were compared to the perennial ryegrass GenomeZipper (Pfeifer *et al.* 2013) in order to obtain the (approximate) locations of the scaffolds of interest on the linkage groups (LGs).

### Genome and gene annotation

To identify transcribed regions of the perennial ryegrass genome and corresponding functional coding DNA sequences (CDS), a variety of RNA-seq datasets were used to predict CDS based on homologous BLAST search and to assign functional descriptions using BLAST homology to reference proteomes and pattern matching algorithms.

#### RNA-seq data:

To identify genic regions and their corresponding introns, exons and splice variants, the Tuxedo suite of tools was used (Trapnell *et al.* 2012). Results from the following RNA sequencing projects were used: six different tissues from *L. perenne* (Bioproject: PRJNA222646; Farrell *et al.* 2014); five *L. multiflorum* datasets from meristem samples (SRR3100250-4; Stočes *et al.* 2016); pollen and stigma samples from *L. perenne* (Manzanares *et al.* 2016); additionally an in-house data set comprising of 48 *L. perenne* meristem samples, taken at 8:00, 16:00 and 00:00 were also included (S. A. Yates, unpublished data). The reads were aligned to the transcriptome using Tophat v2.0.11 and Bowtie2 v2.1.0 (Langmead 2010; Trapnell *et al.* 2012) for all samples. Isoforms of genes were identified using Cufflinks v 2.2.0 (Trapnell *et al.* 2012) producing a genomic feature format file (GFF). The individual GFF files were then merged using the cuffmerge command, default settings.

#### Coding sequence identification:

For CDS identification the spliced exons for each GFF transcript were retrieved using gffread (part of the Tuxedo tool suite). To identify the correct open reading frames (ORF) for protein sequences the program ORFpredictor v3.0 (Min *et al.* 2005) was used. For frame selection, the transcripts were first BLASTX (Altschul *et al.* 1990) searched against a protein database consisting of the proteomes from *Arabidopsis thaliana* TAIR v10 (Swarbreck *et al.* 2008), *O. sativa* (downloaded from Ensembl; Kersey *et al.* 2016), *Glycine max* (Ensembl), *Populus trichocarpa* (Ensembl) and *Manihot esculenta* (v4.1; downloaded from Phytozome; Goodstein *et al.* 2012; Prochnik *et al.* 2012). This database, although not exhaustive, provided a broad basis of existing plant proteins. ORFpredictor was then used to identify CDS by use of the best BLAST hits frame selection. In the absence of a homologous BLAST hit, ORFpredictor selected the longest ORF. These results were then used to annotate the GFF file created by Cufflinks for CDS using scripts kindly provided by Palmieri *et al.* (2012).

#### Gene annotation:

For functional annotation of genes, three synergistic methods were employed, based on protein sequences. First, the protein sequences were search against the *A. thaliana* TAIR10 proteome using BLASTP. Second, the proteins were searched against the Swiss-Prot non-redundant protein database (http://www.uniprot.org/downloads;downloaded03/14/2016; UniProt Consortium 2014), again using BLASTP. In both cases the functional annotation of the best BLAST hit (E≤1e^-15^) protein was used to assign annotations for functional description and gene ontology (GO). From Swiss-Prot an InterPro domain was also assigned where possible. In the third step, the protein sequences were scanned against InterPro’s signatures using InterProScan v5.16-55 (Jones *et al.* 2014). From this, a number of assignments could be made including High-quality Automated and Manual Annotation of Proteins (HAMAP; Pedruzzi *et al.* 2015), Pfam (Finn *et al.* 2016) and Protein Information Resource Super family (PIRSF; Nikolskaya *et al.* 2007). For the aforementioned, the corresponding GO annotation was also retrieved from http://geneontology.org/external2go/ (downloaded 27/06/2016). The three sources of annotation were then combined, using in-house Perl scripts, into a single table and the GO terms from each were concatenated into a non-redundant list. Predicted CDS on the significant scaffolds were annotated using this list.

### Data availability

Figure S1 contains graphs on the number of informative sequence tags per genotype. In Figure S2 the two principal components explaining the greatest variation from a PCA of the genotypic information are plotted. Table S1 provides an overview of the paircross parents and their progeny populations used in this work. Table S2 contains the barcode sequences used for the preparation of the GBS libraries. Table S3 contains gene annotations for all scaffolds found to be significantly associated with the response to anther culture. File S1 contains genotypes and phenotypes of each individual. All deplexed data are available at the Sequence Read Archive (https://www.ncbi.nlm.nih.gov/sra) under BioProject: PRJNA438417. All annotation data are available at http://doi.org/10.5281/zenodo.1208401. Supplemental material available at Figshare: https://doi.org/10.25387/g3.6086876.

## Results

### Phenotypic data

The genotype-dependent response to AC, the wide segregation of androgenic capacity within and the differences between the performance of the bi-parental mapping populations, have been described in detail in Begheyn *et al.* (2017). In addition, a further eighteen genotypes were included in this study (populations 12 and 15; Table S1). A detailed summary of the phenotypic traits can be found in Table 1. A total of 313 and 116 genotypes were subjected to *in vitro* AC in 2015 and 2016, respectively, with an overlap of 78 genotypes between the two years (Begheyn *et al.* 2017). While observations ranged from zero to several hundred or even over 1,000 in the case of plant and green plant production, the majority were zeros (mode = 0) or close to zero (medians; Table 1). As a consequence, all of the eight androgenic capacity-related traits were, even upon transformation, not normally distributed (Begheyn *et al.* 2017), which necessitated the use of nonparametric statistics for the GWAS analyses (Rebaï 1997).

**Table 1:** Summary of the androgenic capacity-related phenotypic traits under study (Begheyn *et al.* 2017). AC – anthers cultured; AP – albino plants; ELS – embryo-like structures; EC – ELS cultured; GP – green plants; RA – responsive anthers

TRAIT	MIN	MAX	MEDIAN	INTERQUARTILE RANGE	NUMBER OF GENOTYPES
2015					
RA (%)	0	86	7.9	27.5	313
ELS per 100 AC	0	665	21	94.9	307
Plants per 100 AC	0	1810	2.4	54	305
Plants per 100 EC	0	800	38.5	95.2	229
GP per 100 AC	0	1530	0	6	297
GP per 100 EC	0	335	0	25	229
AP per 100 AC	0	705	2	28	297
AP per 100 EC	0	800	21.1	52.6	229
2016					
RA (%)	0	87	13	18	116
ELS per 100 AC	0	933	73	117	116
Plants per 100 AC	0	1609	0	9	116
Plants per 100 EC	0	425	0	18.3	105
GP per 100 AC	0	1203	0	0	115
GP per 100 EC	0	318	0	0	104
AP per 100 AC	0	942	0	6.6	115
AP per 100 EC	0	270	0	14.4	104

### Genotyping-by-sequencing (GBS)

Sequencing of the GBS libraries yielded a total of 884,174,849 raw, or 701,662,007 de-multiplexed reads. Of these, 141,775,689 (20.2%) were mapped to the perennial ryegrass genome assembly v1.0 (Byrne *et al.* 2015). After removing non-polymorphic tags (75.6%) and stringent filtering (see Materials and Methods), 1,120 and 1,079 informative tags of 100 bp, containing a polymorphic SNP or haplotype, could be used for the analysis of the 2015 and 2016 datasets, respectively (Figure S1). While the majority contained a single SNP, 25.8% (2015) and 24.2% (2016) of informative tags harbored two or more SNPs. Such sets of SNPs on single tags were treated as haplotypes in subsequent analyses.

Given the multiparental pedigree of the genotypes used in this study, the necessity for applying a correction for population stratification or structure (kinship) was investigated. No evidence for either was found upon analysis of the genotypic data using STRUCTURE (Porras-Hurtado *et al.* 2013), a kinship matrix (VanRaden 2008) or hierarchical clustering. In addition, the two principal components of the PCA explained 76.3% and 10.4% of variation, respectively (Figure S2). It was therefore not deemed necessary to include population structure or relatedness corrections in subsequent analyses.

### Genome-wide association study (GWAS)

Analysis of the 2015 dataset resulted in the identification of significant associations (LOD ≥ 3.0) between six of the studied traits and nine SNPs as well as five haplotypes. Because two of the tags harboring these polymorphisms mapped back to the same scaffold (2554) of the perennial ryegrass genome assembly (Byrne *et al.* 2015), a total of thirteen significant scaffolds were identified (Table 2). No significant associations were found for plant or albino plant regeneration. Analysis of the smaller 2016 dataset yielded seven significant scaffolds (LOD ≥ 3.0) for six traits (Table 2). No significant associations were found for plant production and regeneration and none of the scaffold was significantly associated with a trait in both years given the 3.0 LOD threshold.

**Table 2:** Overview of the significant scaffolds of the perennial ryegrass genome assembly (Byrne *et al.* 2015) detected for each studied trait (LOD ≥ 3.0). Significant differences (*P* ≤ 0.05) between phenotypic medians are indicated with letters. AP – albino plants; AC – anthers cultured; ELS – embryo-like structures; EC – ELS cultured; GP – green plants; LG – linkage groups; RA – responsive anthers

TRAIT	SCAFFOLD	LG	POSITION (cM)	LOD	ALLELE OR HAPLOTYPE	MEDIAN	ALLELE OR HAPLOTYPE	MEDIAN	ALLELE OR HAPLOTYPE	MEDIAN
**2015**										
RA (%)	815	1	33.0-33.3	3.0	C/C	21.0 ^a^	C/T	6.9 ^b^	T/T	6.1 ^b^
	233	4	40.4-40.5	3.9	AC/AC	17.1 ^a^	AC/GT	10.8 ^a^	GT/GT	1.3 ^b^
	16597	4	52.3-52.4	3.4	GAG/GAG	19.6 ^a^	CGA/CGA	5.2 ^b^	CGA/GAG	1.5 ^b^
	1669	5	0	3.2	G/G	14.7 ^a^	G/T	1.3 ^b^		
	2554_2	5	28.5	3.8	C/C	11.7 ^a^	C/T	2.0 ^b^		
	2075	7	43.6-43.7	3.3	GT/GT	19.4 ^a^	TC/TC	13.8 ^a^	GT/TC	1.3 ^b^
	4385	7	46.5	3.1	TG/TG	19.0 ^a^	GA/TG	14.2 ^a^	GA/GA	2.4 ^b^
ELS/100AC	815	1	33.0-33.3	3.4	C/C	73.6 ^a^	C/T	13.1 ^b^	T/T	21.6 ^b^
	233	4	40.4-40.5	3.1	AC/AC	55.9 ^a^	AC/GT	36.9 ^ab^	GT/GT	1.7 ^b^
	16597	4	52.3-52.4	3.9	GAG/GAG	62 ^a^	CGA/CGA	8.3 ^b^	CGA/GAG	2.4 ^b^
	1669	5	0	3.4	G/G	41.9 ^a^	G/T	0.7 ^b^		
	2554_2	5	28.5	4.5	C/C	34.9 ^a^	C/T	3.4 ^b^		
	4385	7	46.5	3.9	TG/TG	54.6 ^a^	GA/TG	32.8 ^a^	GA/GA	0.8 ^b^
	10161	—	—	3.5	C/T	49.7 ^a^	T/T	47 ^a^	C/C	5.0 ^b^
Plants/100AC	16597	4	52.3-52.4	3.0	GAG/GAG	27.0 ^a^	CGA/CGA	0.0 ^b^	CGA/GAG	0.0 ^b^
	2554_2	5	28.5	4.8	C/C	4.9 ^a^	C/T	0.0 ^b^		
	10161	—	—	3.3	C/T	7.9 ^a^	T/T	3.8 ^a^	C/C	0.0 ^b^
GP/100AC	6436	2	79.6-79.8	3.1	T/T	1^a^	C/T	0.0 ^b^	C/C	0.0 ^a^
GP/100EC	3723	5	4.5-25.4	3.1	C/C	64.2 ^a^	C/T	2.0 ^a^	T/T	0.0 ^b^
AP/100AC	16597	4	52.3-52.4	3.2	GAG/GAG	16.3 ^a^	CGA/CGA	0.0 ^b^	CGA/GAG	0.0 ^b^
	2554_1	5	28.5	4.0	G/G	5.8 ^a^	A/G	0.0 ^b^		
	2554_2	5	28.5	5.3	C/C	4.0 ^a^	C/T	0.0 ^b^		
	6186	7	43.6-43.7	3.2	CA/CA	12.7 ^a^	GT/GT	9.7 ^a^	CA/GT	0.0 ^b^
	1607	7	51.6-51.7	3.0	A/A	13.1 ^a^	A/C	6.1 ^a^	C/C	0.0 ^b^
	123	7	62.4-62.8	3.3	G/G	13.8 ^a^	A/G	0.0 ^b^	A/A	0.0 ^b^
**2016**										
RA (%)	8920	4	22.2-22.3	3.4	CC/TT	21.0 ^a^	TT/TT	13.0 ^a^	CC/CC	9.0 ^b^
	15142	5	28.2	3.2	A/G	22.0 ^a^	A/A	11.0 ^ab^	G/G	8.0 ^b^
	60	5	28.5	3.3	T/T	34.0 ^a^	C/T	11.0 ^b^	C/C	8.5 ^b^
ELS/100AC	8920	4	22.2-22.3	3.3	CC/TT	102.0 ^a^	TT/TT	89.0 ^a^	CC/CC	28.5 ^b^
	813	5	28.5-28.5	3.2	A/G	173.0 ^a^	G/G	36.0 ^b^		
GP/100AC	127	1	56.1-57.5	3.9	G/G	1.2 ^a^	A/G	0.0 ^b^	A/A	0.0 ^b^
GP/100EC	127	1	56.1-57.5	4.1	G/G	1.6 ^a^	A/G	0.0 ^b^	A/A	0.0 ^b^
AP/100AC	7045	7	37.5-38.6	3.3	C/C	21.1 ^a^	C/T	0.0 ^b^	T/T	0.0 ^b^
AP/100EC	3194	1	30.9-31.1	3.0	TTTC/TTTC	37.5 ^a^	CCCG/TTTC	0.0 ^b^	CCCG/CCCG	0.0 ^b^
	7045	7	37.5-38.6	3.0	C/C	19.8 ^a^	C/T	0.0 ^b^	T/T	0.0 ^b^

Since non-parametric testing does not allow for an estimation of QTL or allelic effects, allele or haplotype medians per significant scaffold and trait, combined with Dunn’s tests *post hoc* to ascertain significant differences (*P* ≤ 0.05), are presented instead (Table 2). In the 2015 dataset, for example, differences between the medians of the most and least beneficial SNP or haplotype ranged from 9.7 to 18.1 for percentage responsive anthers, 31.5 to 54.2 ELS per 100 AC and 4.9 to 27 plants per 100 AC. The 2016 dataset included a haplotype (TTTC/TTTC) associated with a median albino plant regeneration of 37.5 compared to 0 for the other haplotypes (CCCG/TTTC and CCCG/CCCG) of the same significant scaffold (3194). The smallest significant differences in median, of less than 1 and 1.2 in the 2015 and 2016 datasets, respectively, were observed for green plant production. Nevertheless, for green plant regeneration, the beneficial allele on scaffold 3723 was associated with a median increase of 62.2 green plants per 100 EC compared to the least beneficial allele (Table 2).

**Figure 1:**
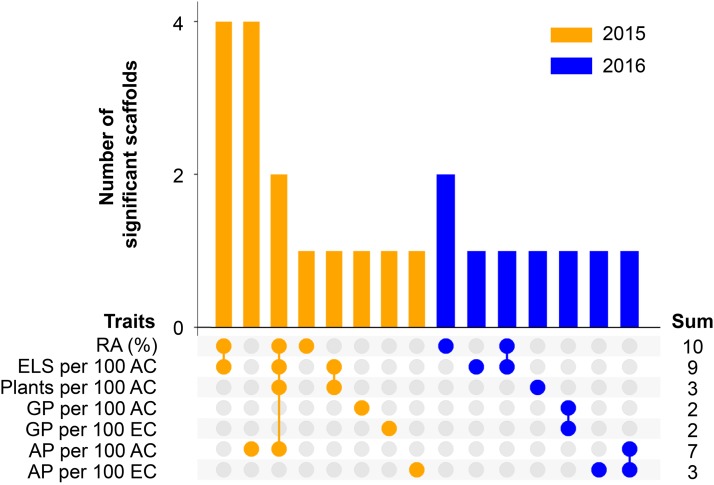
Overview of the number of significant scaffolds per trait or, shown with connected dots, per group of traits (*bars*) and the total number of significant scaffolds per trait (*sum*). AP – albino plants; AC – anthers cultured; ELS – embryo-like structures; EC – ELS cultured; GP – green plants; RA – responsive anthers.

Most significant associations were found for the percentage of responsive anthers (10 associations), embryo production (nine) and albino plant production (seven; Figure 1). Using the 2015 dataset, four scaffolds (815, 233, 1669 and 4385) were significant for both the percentage of responsive anthers as well as ELS production, while two scaffolds (16597 and 2554) were significantly associated with percentage responsive anthers and the production of ELS, plants and albino plants. Scaffold 10616 was significantly associated with ELS and plant production. Three scaffolds, 8920, 127 and 7045 were found to be significant for two traits using the 2016 dataset.

### Positioning significant scaffolds on the GenomeZipper

**Figure 2:**
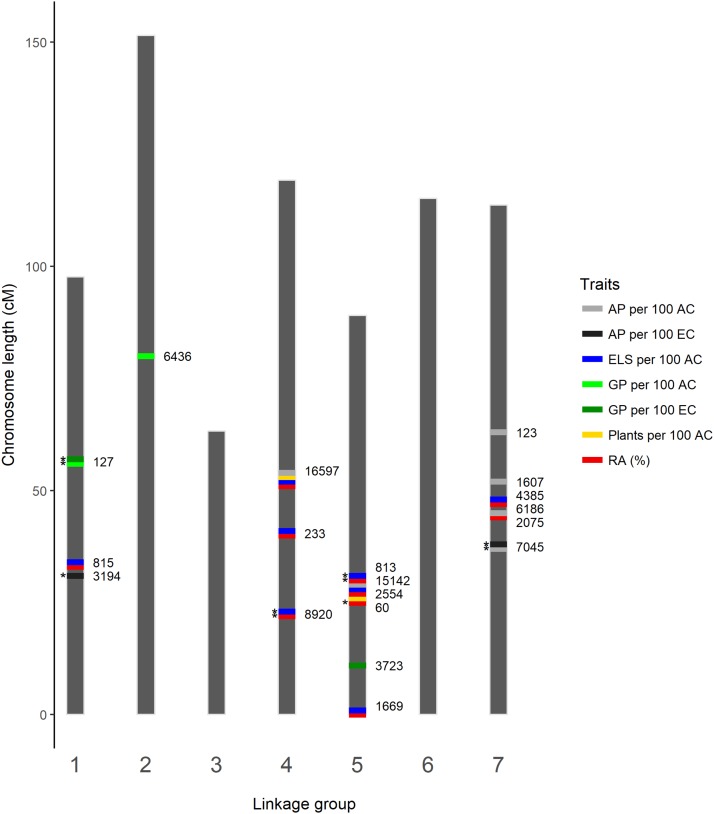
Positions of the significant scaffolds detected in 2015 and 2016 (*) on the perennial ryegrass genome as inferred by the perennial ryegrass GenomeZipper (Pfeifer *et al.* 2013). AP – albino plants; AC – anthers cultured; ELS – embryo-like structures; EC – ELS cultured; GP – green plants; RA – responsive anthers.

By comparing *B. distachyon*, rice and sorghum gene homologs identified on the significant scaffolds with those anchored on the perennial ryegrass GenomeZipper (Pfeifer *et al.* 2013), all but one scaffold could be assigned approximate positions on the LGs (Figure 2). Even so, confidence in the positioning varied from case to case. For example, the approximate positions of scaffolds 123, 127, 233, 813, 2075, 3194, 3723, 6186, 15142 and 16597 were resolved via one or several exact gene matches to the same location on the GenomeZipper. Scaffolds 60, 815, 1607, 1669, 2554, 4385, 6436, 7045 and 8920 were positioned (approximately) using three to 10 genes that were not anchored on the GenomeZipper, but could be placed between several genes anchored at the same location. Scaffold 10616 could not be assigned a location because no significant BLASTN hits of sufficient length were obtained.

Even though no scaffold was found to be significant in both years, scaffolds identified in different years were positioned in similar locations on the GenomeZipper LGs (Figure 2). Scaffolds 815 (2015) and 3194 (2016) are approximately 2 cM apart on LG 1 for example, while scaffolds 60, 813 and 15142 (2016) and 2554 (2015) are all positioned within a 0.3 cM region on LG 5. On the lower middle region of LG 7, scaffolds 2075 and 6186 (43.6 to 43.7 cM) and 4383 (46.5 cM) from the 2015 dataset were positioned in close proximity to each other.

No scaffolds were positioned on LGs 3 and 6. Scaffolds associated with the percentage of responsive anthers, ELS production and at least one of the albino plant-related traits were positioned on LGs 1, 4, 5 and 7, mostly relatively close together. Also amid these, on LGs 4 and 5, were the two plant production-related scaffolds (2554 and 16597) that could be placed on the GenomeZipper. The three scaffolds (127, 3723 and 6463) significantly associated to the green plant-related traits were some distance away from the scaffolds associated to the other traits. In fact, scaffold 6436 was the only scaffold positioned on LG 2.

### Gene annotations

Between one and four predicted genes were annotated for each significant scaffold, with the exception of scaffold 10616 (Table S3). On scaffold 1607 for example, sequence homology to the Arabidopsis *SERRATE* (*SE*) gene was found, while homologs of two domains of Polycomb Repressive Complex 2 (PRC2), *FERTILIZATION-INDEPENDENT ENDOSPERM* (*FIE*) and *CURLY LEAF* (*CLF*) were identified on scaffolds 4383 and 7045, respectively.

## Discussion

Here, we present the first report of genetic loci associated with *in vitro* androgenesis in perennial ryegrass. Between two and 10 QTL (LOD ≥ 3.0) for anther response percentage, embryo production, total plant production as well as green and albino plant production and regeneration were identified on five of the seven perennial ryegrass LGs. Additionally, several intriguing candidate genes that may be responsible for the observed phenotypic differences were predicted on the QTL-harboring scaffolds of the perennial ryegrass genome assembly (Byrne *et al.* 2015). These results enable the development of the first molecular markers for androgenic capacity in perennial ryegrass, from the identified, polymorphic GBS tags. Their availability will help to realize the long-standing aim of efficient, marker-assisted introgression of good responses to *in vitro* DH induction into recalcitrant germplasm (Halberg *et al.* 1990; Andersen *et al.* 1997).

### Multiparental population GWAS in perennial ryegrass

Contrary to previous QTL studies on androgenic capacity, which were based on linkage mapping in bi-parental populations of up to 100 individuals (Muñoz-Amatriaín *et al.* 2008; Krzewska *et al.* 2012; Nielsen *et al.* 2015), an association mapping approach in a multiparental population, composed of 391 heterozygous individuals, was applied here. This design increased the presence of distinct alleles, confirmed by the observed phenotypic variation (Begheyn *et al.* 2017), and, due to the recombination between the nine heterozygous parents, ensured high levels of allelic diversity as well as good mapping resolution (Klasen *et al.* 2012; Giraud *et al.* 2014; Wang *et al.* 2017). Around 1,100 polymorphic SNPs and haplotypes, identified using a methylation-sensitive GBS protocol (Elshire *et al.* 2011), allowed for the genome-wide interrogation of gene-dense regions within the multiparental mapping population (Byrne *et al.* 2015). Significant population structure was absent, due to the common breeding history of the parental plants used to design the mapping population. This powerful experimental design, combined with robust, non-parametric (K-W) single SNP/haplotype genome-wide analysis and permutation-based validation, was successfully used to detect significant QTL (LOD ≥ 3.0) associated with the component traits of the androgenic response of perennial ryegrass.

### A putative major QTL for green plant regeneration on perennial ryegrass LG 5

Authors have often commented on the difficulty of comparing tissue culture experiments, due to highly genotype-specific responses as well as crucial differences in execution and data collection (Bolibok and Rakoczy-Trojanowska 2006; Seldimirova and Kruglova 2015). Fortunately, comparative genomics studies within the grass family allow for an interspecific comparison of cereal AC and IMC QTL studies, albeit at the chromosomal level (Devos 2005). Most homologous grass chromosomes have been associated with all of the androgenicity-related traits at least once, however, and a common pattern is not obvious. One possible exception is a putative locus controlling green plant regeneration, which was identified on Triticeae chromosome group 5 and reported to affect 12–37% of the phenotypic variation in barley, rice (chromosome 9), triticale and wheat (He *et al.* 1998; Torp *et al.* 2001; Chen *et al.* 2007; Muñoz-Amatriaín *et al.* 2008; Krzewska *et al.* 2012). Intriguingly, we identified a putative major QTL, associated with a median increase of 62 green plants per 100 AC, on perennial ryegrass LG 5 as well (Pfeifer *et al.* 2013). This locus is therefore of great interest and its further investigation, for example using fine-mapping approaches, may lead to the identification of the gene with a considerable effect on green plant regeneration in the grass family.

### Genetic control of androgenic capacity

A relatively large number of QTL with modest effects were associated with androgenic traits, such as anther response percentage (10 QTL), embryo production (nine QTL) and albino plant production (seven QTL). In addition, many QTL were shown to affect several traits, confirming the high correlations between, for example, embryo production and anther response as well as plant production observed earlier (Begheyn *et al.* 2017). Similar results have been reported by other groups (Murigneux *et al.* 1994; Beaumont *et al.* 1995; Manninen 2000; Krzewska *et al.* 2012). Finally, QTL detected in 2015 were not detected in 2016 and vice versa, although the QTL identified on scaffold 2075 using the 2015 dataset had a LOD of 2.0 using the 2016 dataset for percentage responsive anthers (results not shown). The discrepancy is probably caused by the fact that only 78 genotypes from four bi-parental crosses were subjected to AC in both years and just 45 of those had the same pair-cross parents (population 1). Allele frequencies of QTL detected using the 2015 dataset were likely too low, or entirely absent, from the 2016 dataset, which in turn harbored distinct beneficial alleles at a high enough frequency for QTL detection. Although a smaller dataset was used in 2016, several QTL of particular interest were detected. For example, a QTL on scaffold 813 was associated with a major median increase in embryo production of 137 ELS per 100 anthers cultured. In addition, the only QTL (on scaffolds 3194 and 7045) associated with albino plant regeneration, connected with an median increase of 19.8 and 37.5 albino plants per 100 ELS cultured, were detected using this dataset.

All of the above findings may be explained by the fact that both ME and albinism during *in vitro* culture are under complex, polygenic and heterogeneous control (Seguí-Simarro and Nuez 2008; Makowska and Oleszczuk 2014). A single genetic master switch for ME has never been identified and albino phenotypes can be caused by mutations in as many as 300 nuclear genes (Kumari *et al.* 2009; Hand *et al.* 2016). A significant increase in embryo production may, therefore, be accomplished via the stacking of several genetic loci with modest effect within single genotypes (Madsen *et al.* 1995; Andersen *et al.* 1997; Marhic *et al.* 1998). In addition to nuclear genes, plastid-encoded genetic factors and their transcription levels have been implicated in the incidence of albinism in Poaceae species during *in vitro* culture (Caredda *et al.* 2004; Torp and Andersen 2009). This not only complicates the elucidation of the genetic control of this phenomenon, but also affects the effectivity of stacking beneficial nuclear genetic factors to achieve lower albinism rates.

A relatively small number of QTL were associated with plant production, green plant production and green and albino plant regeneration. The three QTL detected for total plant production also affected either embryo production, albino production or both. Conversely, the QTL that influenced green plant production (2 QTL) and regeneration (2 QTL) were not associated with any other traits and positioned at distinct locations on the perennial ryegrass LGs. In addition, only one of the two QTL related to albino plant regeneration affected a second trait, albino plant production. These results do not only confirm the separate genetic control of green and albino plant production capacity reported previously (He *et al.* 1998; González *et al.* 2005; Krzewska *et al.* 2015; Begheyn *et al.* 2017). They also suggest that total plant production and total plant regeneration, for which no QTL were identified at all, may not be of great use to describe androgenic ability. The three phases of *in vitro* androgenesis that are commonly distinguished, 1) embryo production, 2) plant regeneration and 3) green plant recovery, can, at least in the grass family, be redefined as 1) embryo production, 2a) green plant recovery and 2b) albino plant recovery. Green plant recovery seems to be controlled by fewer loci than albino plant recovery, although environmental influence on albinism may have masked both green plant production and regeneration capacity as well as the QTL associated with them (Begheyn *et al.* 2017).

### Candidate genes involved in androgenic response

While the putative function of most candidate genes underlying the QTL identified here has yet to be resolved, several have previously been associated with the regulation of stress response, cell fate change, embryogenesis or organogenesis. The *ISOPRENYLCYSTEINE METHEYLESTERASE-LIKE* 2 (*ICME-LIKE2*) gene annotated on scaffold 123, for instance, is involved in abscisic acid (ABA) mediated stress signaling and specifically expressed in reproductive organs of *Arabidopsis* (Lan *et al.* 2010). Similarly, the *VIP HOMOLOG 1* (*VIH1*) gene, identified on scaffold 233, is crucial to certain aspects of jasmonate mediated stress signaling and is mainly expressed in *Arabidopsis* pollen (Laha *et al.* 2015). Phytohormones like ABA and jasmonic acid (JA) have, in fact, been shown to play important roles during androgenesis by ensuring microspore viability through the regulation of stress responses as well as inducing ME via signaling cascades that activate specific gene expression programs (Maraschin *et al.* 2005; Ahmadi *et al.* 2014; Żur *et al.* 2015). The *Arabidopsis SERRATE* (*SE*) gene, which is involved in chromatin modification and microRNA-mediated gene expression regulation during organogenesis, was annotated on scaffold 1607 (Grigg *et al.* 2005; Yang *et al.* 2006). Embryonic lethality and defective post-embryonic organ formation have been reported in *Arabidopsis se* mutants, indicating a possible role for *SE* during plant regeneration after successful ME (Prigge and Wagner 2001; Grigg *et al.* 2005; Lobbes *et al.* 2006).

Most intriguing, however, was the annotation of orthologs to two genes encoding distinct domains of the Polycomb Repressive Complex 2 (PRC2), a highly conserved and important regulator of developmental processes, on scaffolds 4385 and 7045 (Förderer *et al.* 2016). The first, *CURLY LEAF* (*CLF*), encodes one of three SET domain proteins, the others being *MEDEA* (*MEA*) and *SWINGER* (*SWN*), which mediate large-scale chromatin remodelling during embryogenic development (Liu *et al.* 2016). In fact, the mannitol stress treatment used prior to barley IMC was found to induce the upregulation of *CLF* in anther tissue (Muñoz-Amatriaín *et al.* 2009a). The second homolog is a *FIE* domain which is associated with *MEA* in the gametophytic- and endosperm-specific configuration of the PRC2. In *Arabidopsis*, *fie* as well as *clf swn* double mutants are unable to terminate the embryogenic phase of germination and proliferate into so-called PcG callus (Chanvivattana *et al.* 2004; Bouyer *et al.* 2011). Furthermore, the PRC2 complex is involved in the negative regulation of the LEC family as well as *WUS* genes, both of which play key roles in somatic and microspore embryogenesis (Berger *et al.* 2011; Lowe *et al.* 2016). In fact, *LEC1*, *LEC2* and *FUS3* are overexpressed in *clf swn* double mutants of *Arabidopsis* (Makarevich *et al.* 2006). Indeed, *LEC1* (over-)expression was shown to negatively affect ME in both rapeseed and rye (Gruszczyńska and Rakoczy-Trojanowska 2011; Elahi *et al.* 2016). Interestingly, a homolog of the MADS box gene *AGL26*, was annotated along with *FIE* on scaffold 4385. Several MADS box transcription factors, which are key regulators of developmental processes, are negatively regulated by PRC2 as well (Masiero *et al.* 2011). Ultimately, the distinct phases of *in vitro* androgenesis are likely to require different levels of PRC2 mediated repression of specific genes (Förderer *et al.* 2016). Quantification or manipulation of the expression of *CLF*, *FIE*, *AGL26* or any of the other candidate genes during different stages of perennial ryegrass *in vitro* AC could confirm their contribution to successful androgenesis and should determine if and when their expression is beneficial.

### Concluding remarks

Here, we have demonstrated the effectivity of a multiparental genome-wide association mapping approach in perennial ryegrass and report the first genetic loci associated with the response to *in vitro* AC. Elucidation of the exact locations of the QTL detected here will, however, require the availability of a more complete perennial ryegrass genome assembly. It can then be ascertained whether the co-localization of several QTL associated with different traits or detected in different years was, in fact, accurately determined using the GenomeZipper (Pfeifer *et al.* 2013). Future studies on the genetic control of androgenic capacity may then focus on these important regions. Of particular interest is a major QTL for green plant regeneration on LG 5 which, if proven to be effective in different genomic backgrounds, is an excellent candidate for further fine mapping approaches. A second major QTL for embryo production on LG 1 was detected in the smaller of the two datasets that were used here, but nevertheless merits additional investigation. Two of the identified candidate genes, *CLF* and *FIE*, are of great potential interest, given their extensively documented involvement in embryogenesis and organogenesis, although expression studies will have to provide further evidence of their involvement in perennial ryegrass ME (Förderer *et al.* 2016). Presently, our results allow for the development of molecular markers which will enable efficient introgression of androgenic capacity into recalcitrant perennial ryegrass germplasm. The availability of an efficient system for homozygous line production will aid in the establishment of a hybrid breeding system, which should increase the rate of genetic gain in this forage crop of global importance.
